# The Atypical Dopamine Transporter Inhibitor CE-158 Enhances Dopamine Neurotransmission in the Prefrontal Cortex of Male Rats: A Behavioral, Electrophysiological, and Microdialysis Study

**DOI:** 10.1093/ijnp/pyad056

**Published:** 2023-09-19

**Authors:** Claudia Sagheddu, Enzo Cancedda, Farshid Bagheri, Predrag Kalaba, Anna Lisa Muntoni, Jana Lubec, Gert Lubec, Fabrizio Sanna, Marco Pistis

**Affiliations:** Department of Biomedical Sciences, Division of Neuroscience and Clinical Pharmacology, University of Cagliari, Cittadella Universitaria di Monserrato, Monserrato, Cagliari, Italy; Department of Biomedical Sciences, Division of Neuroscience and Clinical Pharmacology, University of Cagliari, Cittadella Universitaria di Monserrato, Monserrato, Cagliari, Italy; Department of Biomedical Sciences, Division of Neuroscience and Clinical Pharmacology, University of Cagliari, Cittadella Universitaria di Monserrato, Monserrato, Cagliari, Italy; Department of Pharmaceutical Sciences, Division of Pharmaceutical Chemistry, Faculty of Life Sciences, University of Vienna, Vienna, Austria; Neuroscience Institute, Section of Cagliari, National Research Council of Italy, Cittadella Universitaria di Monserrato, Monserrato, Cagliari, Italy; Programme for Proteomics, Paracelsus Medical University, Salzburg, Austria; Programme for Proteomics, Paracelsus Medical University, Salzburg, Austria; Department of Biomedical Sciences, Division of Neuroscience and Clinical Pharmacology, University of Cagliari, Cittadella Universitaria di Monserrato, Monserrato, Cagliari, Italy; Department of Biomedical Sciences, Division of Neuroscience and Clinical Pharmacology, University of Cagliari, Cittadella Universitaria di Monserrato, Monserrato, Cagliari, Italy; Neuroscience Institute, Section of Cagliari, National Research Council of Italy, Cittadella Universitaria di Monserrato, Monserrato, Cagliari, Italy; Unit of Clinical Pharmacology, University Hospital, Cagliari, Italy

**Keywords:** ADHD, chronic treatment, cognitive enhancer, dopamine transporter, prefrontal cortex

## Abstract

**Background:**

Dopamine plays a key role in several physiological functions such as motor control, learning and memory, and motivation and reward. The atypical dopamine transporter inhibitor S,S stereoisomer of 5-(((S)-((S)-(3-bromophenyl)(phenyl)methyl)sulfinyl)methyl)thiazole (CE-158) has been recently reported to promote behavioral flexibility and restore learning and memory in aged rats.

**Methods:**

Adult male rats were i.p. administered for 1 or 10 days with CE-158 at the dose of 1 or 10 mg/kg and tested for extracellular dopamine in the medial prefrontal cortex by means of intracerebral microdialysis and single unit cell recording in the same brain area. Moreover, the effects of acute and chronic CE-158 on exploratory behavior, locomotor activity, prepulse inhibition, working memory, and behavioral flexibility were also investigated.

**Results:**

CE-158 dose-dependently potentiated dopamine neurotransmission in the medial prefrontal cortex as assessed by intracerebral microdialysis. Moreover, repeated exposure to CE-158 at 1 mg/kg was sufficient to increase the number of active pyramidal neurons and their firing frequency in the same brain area. In addition, CE-158 at the dose of 10 mg/kg stimulates exploratory behavior to the same extent after acute or chronic treatment. Noteworthy, the chronic treatment at both doses did not induce any behavioral alterations suggestive of abuse potential (e.g., motor behavioral sensitization) or pro–psychotic-like effects such as disruption of sensorimotor gating or impairments in working memory and behavioral flexibility as measured by prepulse inhibition and Y maze.

**Conclusions:**

Altogether, these findings confirm CE-158 as a promising pro-cognitive agent and contribute to assessing its preclinical safety profile in a chronic administration regimen for further translational testing.

**Significance Statement**

The atypical dopamine transporter inhibitor S,S stereoisomer of 5-(((S)-((S)-(3-bromophenyl)(phenyl)methyl)sulfinyl)methyl)thiazole (CE-158) has been recently synthetized and characterized by our laboratories, evidencing promising functional and behavioral effects after systemic acute administration. However, a comprehensive understanding of key features (and possible side effects) associated with a chronic treatment with CE-158 is critical toward a therapeutic strategy based on the enhancement of dopamine neurotransmission via dopamine transporter inhibition. Indeed, psychostimulants are well recognized for their pro-psychotic consequences or abuse liability, making them unsuitable for clinical approval. We present here an investigation in adult male rats repeatedly administered with CE-158 at different doses. Under our conditions, CE-158 proved no evidence of inducing psychotic signs or abuse liability, whereby it corroborated its prefrontal-related pro-active efficacy. Our findings support the safety profile of CE-158 as a promising agent for the treatment of pathological conditions, such as attention deficit hyperactivity disorder or dementia, characterized by altered prefrontal dopamine neurotransmission.

## INTRODUCTION

Dopamine plays a key role in several physiological functions such as motor control, reproductive behaviors, learning and memory, and motivation and reward (Beaulieu and Gainetdinov, 2011; Klein et al., 2019; Melis et al., 2022). The central role of dopamine in these functions is highlighted by the fact that alterations in dopamine neurotransmission lead to several neurological and/or neuropsychological disturbances, such as Parkinson disease, schizophrenia, mood disorders, attention deficit hyperactivity disorder (ADHD), and substance use disorders (Castellanos and Tannock, 2002; Nestler et al., 2002; Maia and Frank, 2017; Volkow et al., 2017; de Natale et al., 2018).

In this regard, the membrane dopamine transporter (DAT) represents a privileged target for the development of new classes of drugs (German et al., 2015), being a key factor in regulating synaptic dopamine in both physiological and pathological contexts (Kuhar et al., 1990; Gainetdinov and Caron, 2003; Leo et al., 2018; Sanna et al., 2020). Accordingly, the atypical DAT inhibitor modafinil has been approved for the treatment of sleeping disturbances and narcolepsia. Modafinil has also been proposed for the treatment of neurological and psychiatric conditions (e.g., Alzheimer disease or ADHD) as well as drug addiction (Mereu et al., 2013) and also as a cognitive enhancer in healthy people (Repantis et al., 2010; Battleday and Brem, 2015). However, in this latter case, there are some important ethical considerations (Brühl et al., 2019). Although it shares a common mechanism of action with other psychostimulants such as cocaine and methamphetamine, modafinil has conversely shown low abuse liability (Wood et al., 2014; Bisagno et al., 2016) due to its reduced interference with mesolimbic dopamine. Accordingly, preclinical studies with modafinil and modafinil analogues displayed an improved circuit-related selectivity of these new classes of compounds (Mereu et al., 2017; Sagheddu et al., 2020).

Among novel modafinil analogues, CE-123 has been found effective in enhancing the motivational tone (Rotolo et al., 2019), increasing memory performance (Kristofova et al., 2018; Lubec et al., 2023), and improving behavioral flexibility and impulsivity control (Nikiforuk et al., 2017), with low abuse liability (Sagheddu et al., 2020). Similarly, a high-affinity analogue, S,S stereoisomer of 5-(((S)-((S)-(3-bromophenyl)(phenyl)methyl)sulfinyl)methyl)thiazole (CE-158), demonstrated the ability to increase motivational tone in a model of impaired effort-related behavior, improve social memory and behavioral flexibility, and reinstate hippocampal synaptic plasticity in the aging brain (Kalaba et al., 2020; Rotolo et al., 2020; Lubec et al., 2021; Ebner et al., 2022).

The prefrontal cortex (PFC) represents a brain area of particular interest for the actions of these compounds as here dopamine is directly involved in higher-order cognitive processes and executive functions (Robbins and Arnsten, 2009; Spencer et al. 2015) by acting on different neuronal populations, in particular pyramidal neurons and GABAergic interneurons, through local circuits and projections to other cortical and subcortical brain regions (Anastasiades and Carter, 2021; Howland et al., 2022). Dysfunctions at these networks are at the basis of peculiar symptoms of pathological conditions such as ADHD, schizophrenia, dementia, drug addiction, and a state often referred to as “hypofrontality.” Hypofrontality is characterized by impulsive-compulsive behaviors and impairments in working memory and attentive and preattentive processes such as sensorimotor gating, stereotypies, and perseverations.

The present study aims at (1) investigating the ability of CE-158 to potentiate dopamine neurotransmission at the level of the PFC by means of intracerebral microdialysis and in vivo electrophysiology; and (2) investigating the behavioral effects of CE-158 after acute and chronic treatment on PFC-related behaviors, with particular attention to potential side effects due to the chronic administration.

## METHODS

### Subjects

Male Sprague-Dawley rats (Harlan Nossan, San Pietro al Natisone, Italy), weighing 250–300 g, were kept in groups of 4 in standard cages under controlled environmental conditions (22°C ± 2°C, 60% humidity, 12-hour-light/-dark cycle, with lights on from 7:00 am to 7:00 pm), with water and standard laboratory food ad libitum. All the experiments were performed between 10:00 am and 6:00 pm according to the guidelines of the European Communities Directive (2010/63/EU) and the Italian Legislation (D.P.R. 116/92) and were approved by the Ethical Committee for Animal Experimentation of the University of Cagliari.

### Drugs

CE-158 was synthetized as previously described (Lubec et al., 2021). Kolliphor EL was purchased from Sigma Aldrich (Sigma-Aldrich Chemie GmbH, Germany). All the other reagents were from commercial sources and of the highest purity available.

### Experimental Design

According to the 3R principles governing animal experimentation, all possible effort was made to minimize animal suffering and reduce the number of animals used. Four cohorts of adult male rats were divided in 3 treatment groups: controls (30% Kolliphor EL in physiological solution, 1 mL/kg), 1 mg/kg mg/kg CE-158, and 10 mg/kg CE-158. Rats were administered i.p. once a day for 10 consecutive days according to their treatment group. The first cohort was used for the microdialysis experiments that were performed at the 10th day of treatment as described below. The second cohort was used to perform the electrophysiology experiments. In this case, on the last day of treatment, one-half of them, belonging to the 3 treatment groups, were directly used within 45–60 minutes after the last administration for the electrophysiological recordings into the mPFC. The second half was used for dose-curve experiments, and in this case incremental doses of CE-158 were injected i.v. to 10 mg/mL/kg cumulative dose during the electrophysiology recordings. The third cohort was used to perform the behavioral experiments on exploration, locomotion, and grooming that were performed in the same animals after the first and last treatments at day 1 and day 10, respectively. Finally, the fourth cohort was devoted to the Y-maze and PPI experiments that were performed in the same animals after the first (day 1) and last (day 10) treatments. Regarding behavioral studies and according to the microdialysis results, rats received the i.p. treatment with CE-158 or vehicle 30–45 minutes before the beginning of the test.

### Microdialysis Experiment for Determination of Extracellular Dopamine in mPFC

The intracerebral microdialysis for the determination of dopamine content in the mPFC of rats treated with CE-158 or vehicle was performed in awake, freely moving animals as already described (Sanna et al., 2015, 2017). The day before the experiment, rats were positioned in a stereotaxic apparatus (Stoelting Co., Wood Dale, IL, USA) and, under isoflurane anesthesia (1.5%–2%) (Harvard Apparatus, Holliston, MA, USA), were implanted with a vertical microdialysis probe with a dialysis membrane of approximately 2–3 mm of free surface directed unilaterally at the mPFC (PrL and IL; coordinates: 3.0 mm anterior and 0.7 mm lateral to bregma, and 5.5 mm ventral to dura) according to Paxinos and Watson (2004). The day of the experiment, the animals were transferred to a sound-proof room and after a 1-hour habituation period, the microdialysis probe was connected with polyethylene tubing to a CMA/100 micro-infusion pump (Harvard Apparatus, Holliston, MA, USA) and perfused with a Ringer’s solution (147 mM NaCl, 3 mM KCl, and 1.2 mM CaCl_2_, pH 6.5) at a flow rate of 2.5 µL/min. After an equilibration period of 2 hours of the perfusion medium with the extracellular fluid, dialysate aliquots of 37.5 µL were collected every 15 minutes in polyethylene tubes kept on ice. After the collection of 4 aliquots, rats were administered i.p. with 1 or 10 mg/kg CE-158 or vehicle, and other 8 dialysate aliquots were collected every 15 minutes. Dopamine concentrations in the dialysates were measured by high-pressure liquid chromatography on a 7.5-cm × 3.0-mm i.d., Supelcosil C18, 3-µm-particle size column (Supelco, Supelchem, Milan, Italy) coupled to electrochemical detection (Coulochem II, ESA, Cambridge, MA, USA) using a 5011 dual cell. Detection was performed in reduction mode, with potentials set to +350 and −180 mV. The mobile phase was 0.06 M citrate/acetate pH 4.2, containing methanol 20% v/v, 0.1 mM EDTA, 1 µM triethylamine, and 0.03 mM sodium dodecyl sulphate at a flow rate of 0.6 mL/min. The sensitivity of the assay was 0.125 pg. For the procedures related to the histological verification of probe placement, see supplementary Figure 1.

### In Vivo Single-Unit Electrophysiological Recordings

Rats were anesthetized with 400 mg/kg i.p. chloral hydrate and placed in the stereotaxic frame. Single neurons located in layers III–VI of prelimbic/infralimbic cortex (AP: +2.8–3.6 mm from bregma, L: 0.8–1.0 mm from the midline, V: 1.5–4.0 mm from the cortex) was recorded with glass micropipettes filled with 0.5 M sodium acetate. Individual action potentials were isolated and amplified (1- to 10,000-Hz filter) by means of a CP511AC amplifier (Grass Instruments Co., Quincy, MA, US). Experiments were sampled with Spike2 software by a CED1401 interface (Cambridge Electronic Design, Cambridge, UK). Two types of neurons were isolated and recorded based on waveform shape and frequency of action potentials. Putative pyramidal neurons were selected in accordance with biphasic positive–negative deflections and >2-ms-wide action potentials, regular or irregular activity (Connors and Gutnick, 1990; Au-Young et al., 1999; Secci et al., 2019). Putative GABA-interneurons were identified according to biphasic negative–positive deflections in accordance with single-unit recordings from other brain regions (Steffensen et al., 1998; Schwaller et al., 2004). In such experimental settings, all types of neurons exhibited a <10-Hz action potential discharge rate. To estimate the general activity, the electrode was passed in 4–6 tracks, and the number of active cells was divided by the number of tracks. Spontaneous firing rate and firing regularity (expressed by coefficient of variation, CV, the SD of interspike intervals divided by the mean interspike interval) were determined.

For dose-curve experiments, CE-158–induced changes in firing frequency were calculated by averaging the effects for the 2-minute period following acute i.v. administration of each dose.

### Locomotor Activity, Rearing, and Grooming

The day before the first experiment session (i.e., the day before acute treatment) rats underwent two 1-hour habituation sessions to prevent the influence of novelty factors linked to the experimental procedure and motility apparatus (Angioni et al., 2016). To this aim, rats were transferred to a soundproof room with a light level of 30 lux and positioned in individual cages for habituation. The day of the experiment, rats were transferred in the same experimental room and, after 1 hour, CE-158 or vehicle was administered. Rats were individually tested with a Digiscan Animal Activity Analyzer (Omnitech Electronics, Columbus, OH, USA). Each cage (42 cm × 42 cm × 63 cm) had 2 sets of 16 photocells located at right angles to each other, projecting horizontal infrared beams 2.5 cm apart and 2 cm above the cage floor. Horizontal activity was measured from 30 to 90 minutes after the treatment (for a total of 60 minutes) as total number of sequential infrared beam breaks (counts) in the sensors, recorded every 5 minutes, beginning immediately after placing the animals into the cage. Rearing and grooming episodes were counted by 2 independent observers unaware of the treatment conducted on videotape recordings. Rearing episodes were counted when the rat stretched vertically on its hind legs and exhibited the classic sniffing behavior of the surrounding environment. Grooming behavior was defined as reported in Berridge and colleagues (2005).

### PPI of the Startle Response

Rats were tested as previously described (Noli et al., 2017; Sagheddu et al., 2021). The apparatus (Med Associates) consisted of 4 standard cages placed in sound-attenuated chambers with fan ventilation. Each cage consisted of a Plexiglas cylinder (5 cm diameter) mounted on a piezoelectric accelerometric platform connected to an analog-digital converter. Two separate speakers conveyed background noise and acoustic bursts, each one properly placed to produce a variation of sound within 1 dB across the startle cage. Before each testing session, acoustic stimuli and mechanical responses were calibrated. The testing session featured a background noise of 70 dB and consisted of an acclimatization period of 5 minutes, followed by 3 consecutive sequences of trials (blocks). Unlike the first and the third block, during which rats were presented with only 5 pulse-alone trials of 130 dB, the second block consisted of a pseudorandom sequence of 50 trials, including 12 pulse-alone trials; 30 trials of pulse preceded by 68-, 70-, or 80-dB prepulses (10 for each level of prepulse loudness); and 8 no-stimulus trials, where only the background noise was delivered. Inter-trial intervals were randomly selected between 10 and 15 seconds. The percent PPI value was calculated using the following formula: 100 − [(mean startle amplitude for prepulse pulse trials/mean startle amplitude for pulse-alone trials)*100]. For the analyses of data presented here, PPI values related to different prepulse intensities were collapsed, given that no significant differences among them were observed.

### Y-Maze

On the day of the test, rats were transferred to a soundproof room and left in their home cages for 30 minutes for habituation. Then they were treated with CE-158 or vehicle and after 30 minutes positioned in a Y-maze. Rats were randomly placed at the end of 1 arm of the Y-maze, and the sequence of arm entries was recorded for 10 minutes. The number and sequence of arm entries referred to the 10-minute test were counted for each rat and analyzed by 2 independent observers unaware of the treatment done on videotape recordings. An arm visit was recorded when a rat moved all 4 paws into the arm. An alternation was defined as consecutive entries into all 3 arms (e.g., 1, 2, 3 or 1, 3, 2). The number of maximum alternations was the total number of arm entries minus 2, and the percentage of alternations was calculated as the ratio of actual to maximum alternations multiplied by 100: (actual alternations/maximum alternations)*100 (see for instance, Carton et al., 2021).

### Data Analysis and Statistics

All data are given as mean ± SEM and are expressed as absolute values or percentages.

For in vivo electrophysiology, statistical significance was assessed using parametric 1-way ANOVA. Contingency of population was analyzed by chi-squared test.

For in vivo microdialysis, raw data were percent transformed (with 100% as the average of the last 4 dopamine basal values before treatment), and statistical analyses were performed with 2-way repeated-measures (RM)-ANOVAs using treatment as the between-subjects factor and time (i.e., dialysate fractions) as the within-subjects factor.

For the behavioral tests, 2-way RM-ANOVAs using treatment as the between-subjects factor and time (i.e., duration of treatment or time fraction during the experiment) as the within-subjects factor were performed.

When ANOVA revealed statistically significant main effects or interactions, pairwise comparisons were performed by using Bonferroni corrected paired *t* tests or the Tukey multicomparison test, respectively.

All analyses were performed by using the software GraphPad Prism 8. The significance level was set at *P* < .05.

## RESULTS

### Microdialysis in mPFC for Determination of Extracellular Dopamine

**Figure 1.**
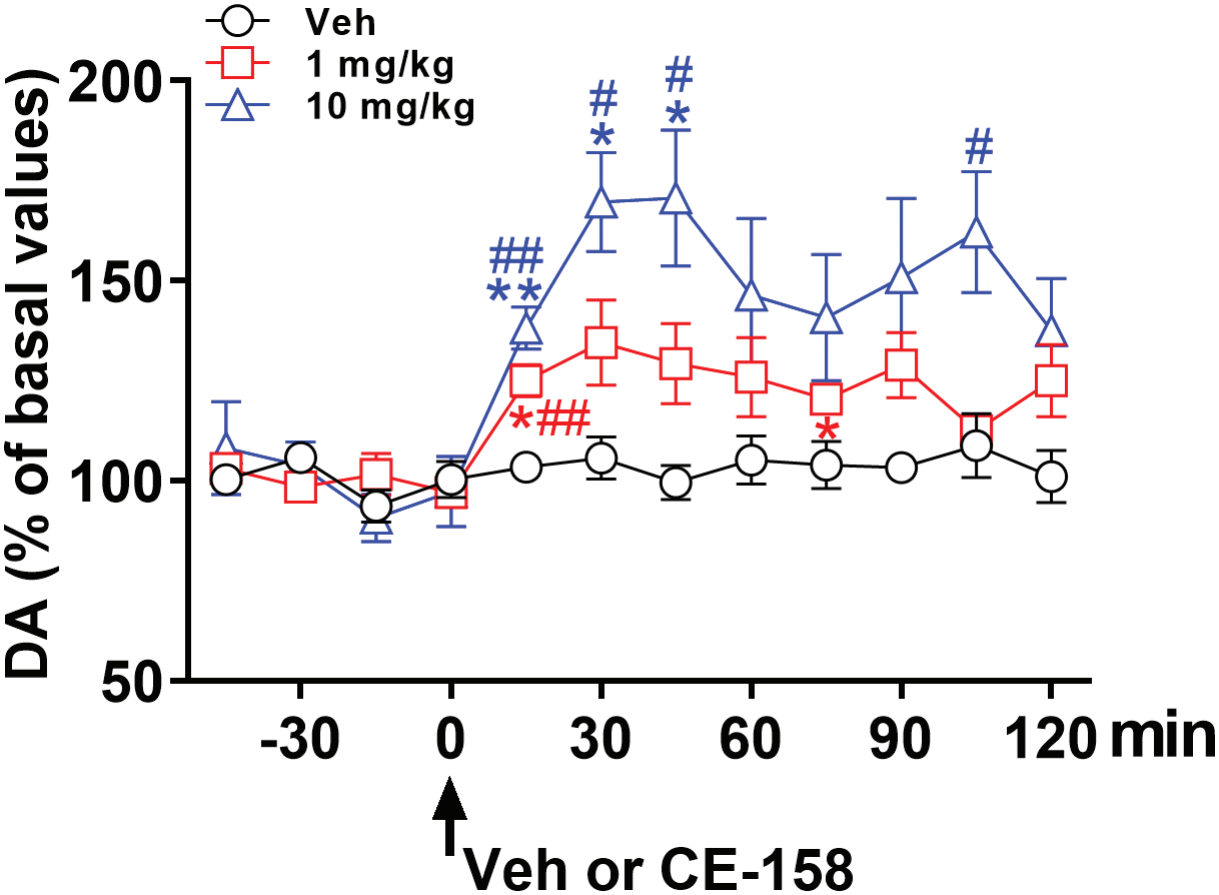
Extracellular dopamine (DA) concentrations in the medial prefrontal cortex (mPFC) dialysates obtained from rats treated with CE-158 (1 or 10 mg/kg, i.p.) or vehicle (Veh). Data are reported as percentages (with 100% as the average of the last 4 dopamine basal values before treatment) with values expressed as means ± SEM of 5 rats per group. *P < .05 compared with basal values (before treatment); ^#^*P* < .05 with respect to vehicle-treated rats (2-way RM-ANOVA followed by Tukey multiple-comparison test).

The first goal of our investigation was to assess the ability of chronic treatment with CE-158 to stimulate dopamine neurotransmission in the mPFC, similar to that seen in other brain areas (Lubec et al., 2021; Ebner et al., 2022). To this aim, we measured extracellular dopamine levels in the mPFC by means of intracerebral microdialysis following systemic administration of 1 or 10 mg/kg CE-158. Basal extracellular dopamine amounts in the mPFC did not differ among the 3 treatment groups and were approximately 2.20 pg in 20 μL of dialysate, corresponding to an extracellular dopamine concentration of approximately 0.70 nM. As shown in Figure 1, CE-158 produced a dose-dependent increase in extracellular dopamine concentrations up to 30% and 70% above basal values, for 1 and 10 mg/kg, respectively (2-way ANOVA, dose*time interaction: F_(22,132)_ = 2.73; dose: F_(2,12)_ = 14.16; both *P* < .001). Moreover, Tukey post hoc comparisons indicated that although CE-158 is effective in increasing dopamine content in the mPFC already 15 minutes after the treatment, the peak is detectable at 30 minutes, with the effect lasting for at least 75–105 minutes (see Figure 1 for single points of statistical significance).

### In Vivo Single-Unit Extracellular Recordings From the mPFC

**Figure 2.**
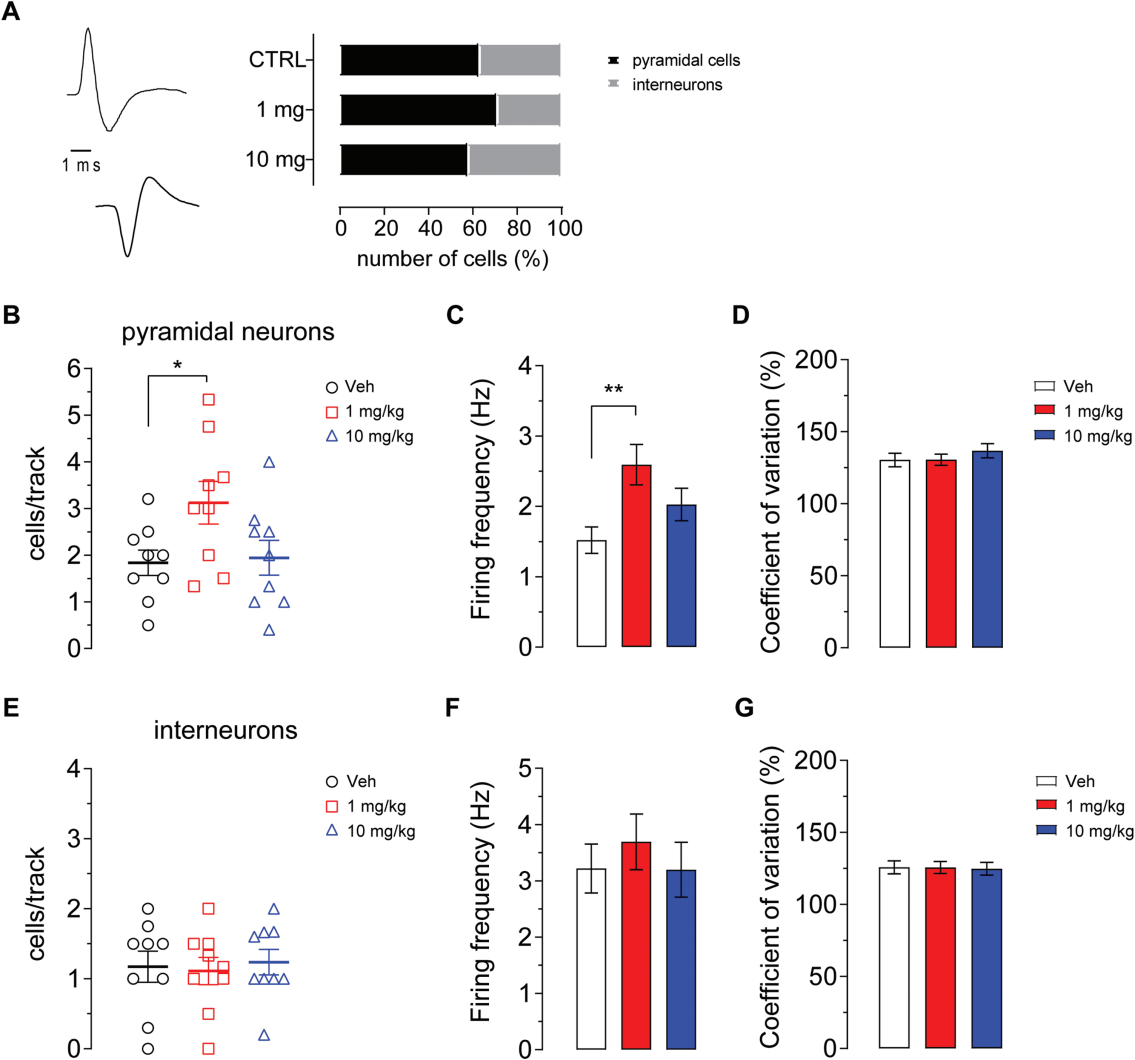
Typical action potential of pyramidal neuron (A, top) and γ-aminobutyric acid (GABA) interneuron (A, bottom) extracellularly recorded from the medial prefrontal cortex (mPFC). Histograms showing the percentage of pyramidal neurons and GABA interneurons from the mPFC of the 3 experimental groups (A, right). Number of mean active pyramidal neurons from the mPFC of the 3 experimental groups (B). Mean firing frequency (C) and coefficient of variation (D) of pyramidal neurons from the mPFC of the 3 experimental groups. Number of mean active interneurons from the mPFC of the 3 experimental groups (E). Mean firing frequency (F) and coefficient of variation (G) of interneurons from the mPFC of the 3 experimental groups. Values are expressed as mean ± SEM. *P < .05; **P < .01 compared with vehicle (Veh)-treated rats (1-way ANOVA followed by Bonferroni multiple comparison test).

We previously showed that acute administration of CE-158 activates in vivo pyramidal neurons from the mPFC of young and old rats (Lubec et al. 2021). Here, we extended the functional analysis of the electrophysiological properties of putative pyramidal neurons (Figure 2A, top left) and GABA interneurons (Figure 2A, bottom left) following chronic treatment with CE-158 for 10 days or vehicle as control. We recorded 50 pyramidal and 30 GABA cells from controls (n = 9 rats), 66 pyramidal and 27 GABA cells from rats treated with 1 mg/kg (n = 9), and 49 pyramidal and 35 GABA cells from rats treated with 10 mg/kg (n = 9). A χ^2^ test revealed that CE-158 did not change the population of active neurons among groups (χ^2^_(2)_ = 3.41, *P* = .18; Figure 2A, right).

Chronic treatment with 1 mg/kg CE-158 increased the number of spontaneously active cells (3.12 ± 0.46 cells per track) (Figure 2B; 1-way ANOVA F_(2,24)_ = 3.6; *P* = .043) compared with control treatment (1.837 ± 0.271 cells per track) and with high-dose treatment (1.942 ± 0.373 cells per track).

Moreover, analysis of firing activity showed an increased firing frequency (Figure 2C; 1-way ANOVA F_(2,161)_ = 4.719; *P* = .010) by CE-158 1 mg/kg (2.59 ± 0.29 Hz; ctrl: 1.52 ± 0.19 Hz; 10 mg/kg: 2.03 ± 0.23 Hz) but no change in the pattern as measured by the coefficient of variation (Figure 2D; 1-way ANOVA F_(2,161)_ = 0.613; *P* = .543) in the 3 groups (ctrl: 130.5 ± 4.72%; 1 mg/kg: 130.6 ± 3.92%; 10 mg/kg: 136.8 ± 4.9%).

Neither the number of spontaneously active interneurons (Figure 2E; 1-way ANOVA F_(2,24)_ = 0.098 *P* = .91) nor the firing frequency (Figure 2F; 1-way ANOVA F_(2,89)_ = 0.33, *P* = .72) or pattern (Figure 2G; 1-way ANOVA F_(2, 89)_ = 0.017, P = .98) was altered by chronic treatment with 1 mg/kg CE-158 (firing 3.69 ± 0.49 Hz, CV 125.7 ± 4.16 %) or 10 mg/kg CE-158 (firing 3.19 ± 0.49 Hz, CV 124.8 ± 4.41%) compared with its vehicle (firing 3.22 ± 0.46 Hz, CV 125.9 ± 4.50 %).

**Figure 3.**
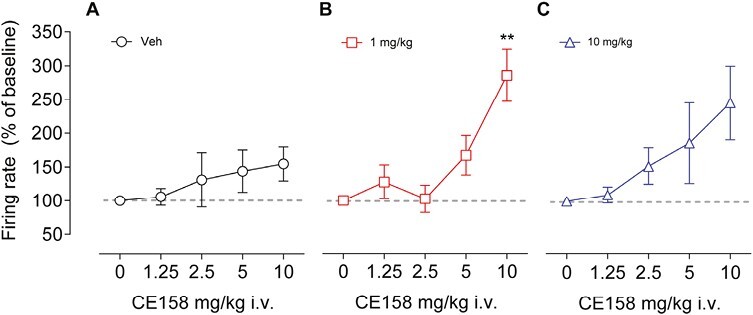
Mean firing rate of medial prefrontal cortex (mPFC) pyramidal neurons following cumulative doses of CE-158 intravenously (i.v.) administered in rats pretreated for 10 days with vehicle (Veh) (A), 1 mg/kg (B), or 10 mg/kg (C). Values are expressed as mean ± SEM. **P < .01 compared with the matched dose of the vehicle-pretreated rats (RM 2-way ANOVA followed by Bonferroni multiple comparison test).

Cumulative dose-response curves to CE-158 (1.25–10 mg/kg i.v.) were also performed to compare its effect on pyramidal neuron activity at the end of the 10 days of chronic exposure. In vehicle-pretreated rats, acute administration of CE-158 elicited a plateau in firing rate (Figure 3A), while in rats pretreated with the lower dose (Figure 3B) the frequency significantly augmented at the maximal cumulative dose (RM 2-way ANOVA, F_(4, 48)_ = 3.57, *P* = .012). Finally, in rats pretreated with the higher dose (Figure 3C), acute injection of CE-158 increased pyramidal neuron firing in a dose-dependent fashion.

### Exploratory Behavior

**Figure 4.**
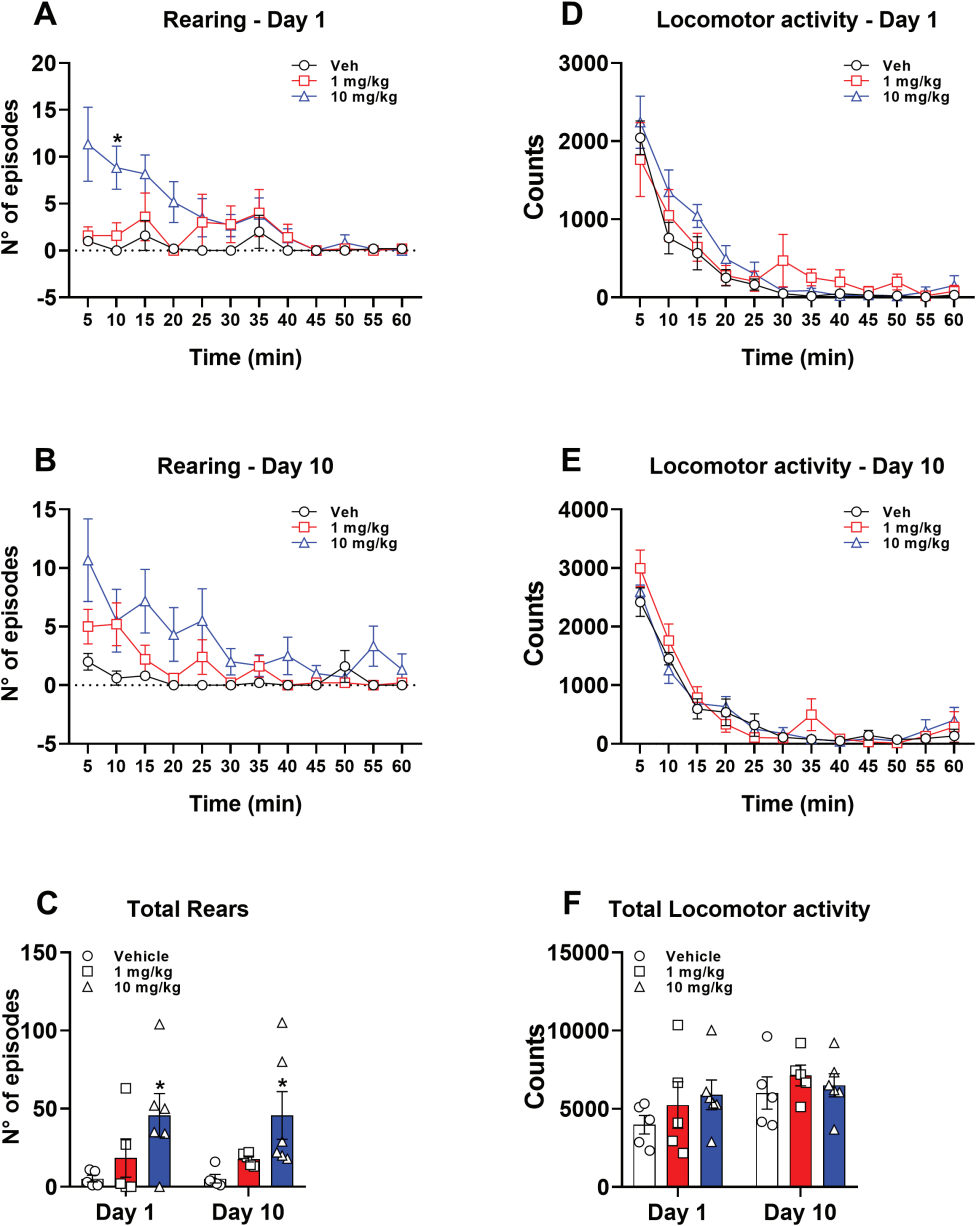
Rearing episodes and horizontal locomotor activity in rats treated with CE-158 1 mg/kg (n = 5) or 10 mg/kg (n = 6) i.p. compared with vehicle (Veh)-treated rats (n = 5) on day 1 and day 10 of the treatment. Figure 4A and 4B indicate the number of rearing episodes and Figure 4D and 4E the counts for horizontal locomotor activity during the test relative to the 5 minutes (min) fractions. In Figure 4C and 4F are reported the values for the entire 60 minutes test at day 1 and day 10. Values are expressed as mean ± SEM. *P < .05 compared with vehicle-treated rats (RM 2-way ANOVA followed by Bonferroni multiple comparison test).

Exploratory behaviors are widely acknowledged as suggestive of general well-being in rodents, whereas diminished locomotion and rearing have been associated with stress and discomfort (Pisula and Siegel, 2005; Sturman et al., 2018). Accordingly, typical exploratory and interactive behaviors could be affected by dopamine dysregulations, which may result from both pathological conditions and disruptive drugs (Arenas et al., 2016). As reported in Figure 4, CE-158 dose-dependently increased the number of rearing episodes to the same extent after the first (i.e., acute) and the last drug administration (i.e., after 10 days of treatment). The number of rearings, although with some differences between the 2 doses, was higher during the first 30 minutes after the positioning of the animal into the experimental cage and tended to decrease towards control values in the second 30 minutes, both at day 1 (Figure 4A) and day 10 (Figure 4B) of treatment. Accordingly, RM 2-way ANOVA detected a significant effect of time (F_(4.14,5.53)_ = 5.533, *P* < .001) and a significant time*treatment interaction (F_(22,143)_ = 2.932, *P* < .0001) for day 1 of treatment and a significant effect of time (F_(3.89,5.55)_ = 5.55, *P* < .001) and of treatment (F_(2,13)_ = 4.323, *P* < .05) for the test performed after the last treatment at day 10. The efficacy of the compound in stimulating the exploratory behavior after chronic treatment was also confirmed by the analyses performed on the total number of rearing episodes at day 1 and day 10 of treatment (Figure 4C). Accordingly, 2-way ANOVA detected a significant effect of the treatment (F_(2,13)_ = 9.527, *P* < .01) but not of the day or a significant day*treatment interaction. Moreover, post hoc comparisons revealed that on both day 1 and 10 of treatment, the rats treated with the dose of 10 mg/kg CE-158, but not those with 1 mg/kg, displayed a significantly higher number of rearing episodes compared with vehicle-treated rats. Taken together, these results indicate that CE-158 dose- and time-dependently induces an increase in exploratory behavior and that this effect is maintained without significant changes over a period of chronic treatment of 10 days.

Chronic treatment with psychostimulants can induce exaggerated locomotor activity and behavioral sensitization, which is considered a sign of the addictive effects of a drug (Everitt and Wolf, 2002). Hence, we also investigated if the chronic administration regimen of CE-158 used here was able to induce this effect. To this aim, we measured horizontal locomotor activity in cages equipped with infrared motion detectors. As expected, and similarly to what was seen with rearings (see above), over the test, rats reduced horizontal movements (time, day 1: F_(2.19,28.49)_ = 48.71, *P* < .0001; Figure 4D; day 10: F_(3.94,51.17)_ = 91.64, *P *< .0001; Figure 4E) but in this case with no difference among treatment groups at both day 1 (treatment: F_(22,143)_ = 1.073, *P* = .382; Figure 4D) and day 10 (treatment: F_(22,143)_ = 1.145, *P* = .307; Figure 4E). Furthermore, no difference was observed in the total locomotor activity referred to the entire 60-minute test among the 3 treatment groups, at both day 1 and day 10 (2-way ANOVA, F_(2,13)_ = 0.684, *P* = .52; Figure 4F), although a general increase in the total counts was observed in the 3 treatment groups passing from day 1 to day 10, regardless the treatment received (2-way ANOVA, main effect of day: F_(1,13)_ = 7.48, *P *< .05; Figure 4F). These results indicate that subchronic treatment with CE-158 at 1 and 10 mg/kg does not induce behavioral sensitization, in contrast to classical psychostimulants such as cocaine and amphetamine (Everitt and Wolf, 2002).

### PPI

Dopaminergic psychostimulants can solicit abnormalities in sensorimotor gating relevant to psychotic events and schizophrenia (Lapworth et al., 2009; Fiorentini et al., 2021). To exclude possible pro-psychotic side effects of the novel compound CE-158, we performed the PPI of the acoustic startle reflex test, a reliable tool for the assessment of sensorimotor gating acknowledged for high translational significance (Geyer et al., 2001; Mena et al., 2016), as a probe of proper PFC functioning under chronic dopamine stimulation by CE-158 (Shoemaker et al., 2005; Mena et al., 2021).

**Figure 5.**
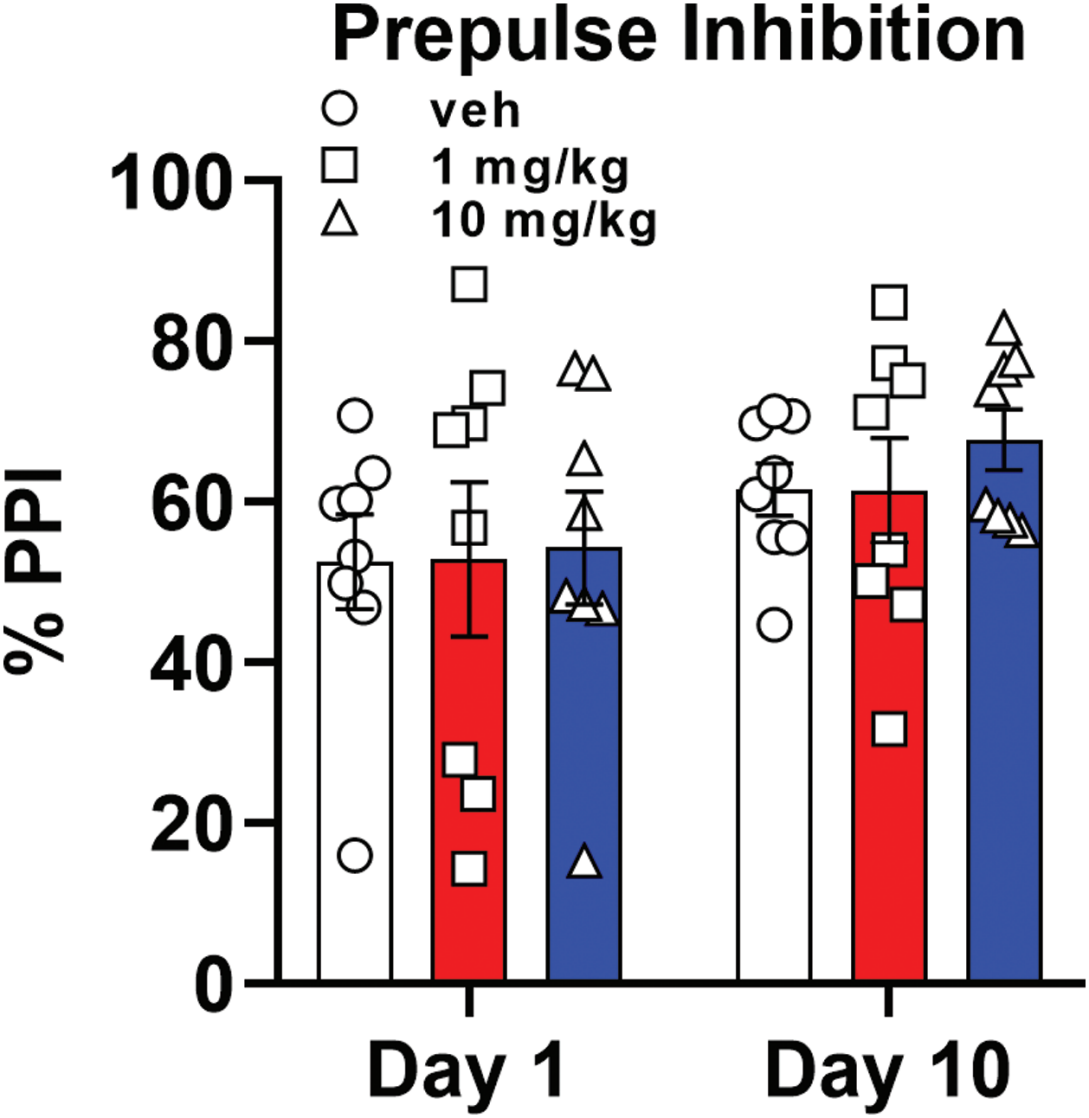
Assessment of prepulse inhibition (PPI) of the startle response in rats treated with CE-158 1 mg/kg or 10 mg/kg compared with vehicle (Veh)-treated rats after the first (day 1) and the last (day 10) treatment. Values are expressed in percentages (see Materials and Methods section) and are mean ± SEM of 8 rats/group (RM 2-way ANOVA: not significant).

As reported in Figure 5, neither acute nor chronic treatment of rats with 1 or 10 mg/kg CE-158 affected PPI values (2-way ANOVA, F_(2,21)_ = 0.175, *P* = .84) compared with vehicle-treated controls. Similarly to locomotor activity, a slightly significant increase in PPI values was observed for all 3 groups regardless of the treatment received passing from day 1 to day 10 (2-way ANOVA, F_(1,21)_ = 7.14, *P* < .05). Seemingly, CE-158 at the tested doses and over a period of 10 days of treatment did not induce impairment or disruption of sensorimotor gating as potential anticipation towards psychosis.

### Y-Maze

**Figure 6.**
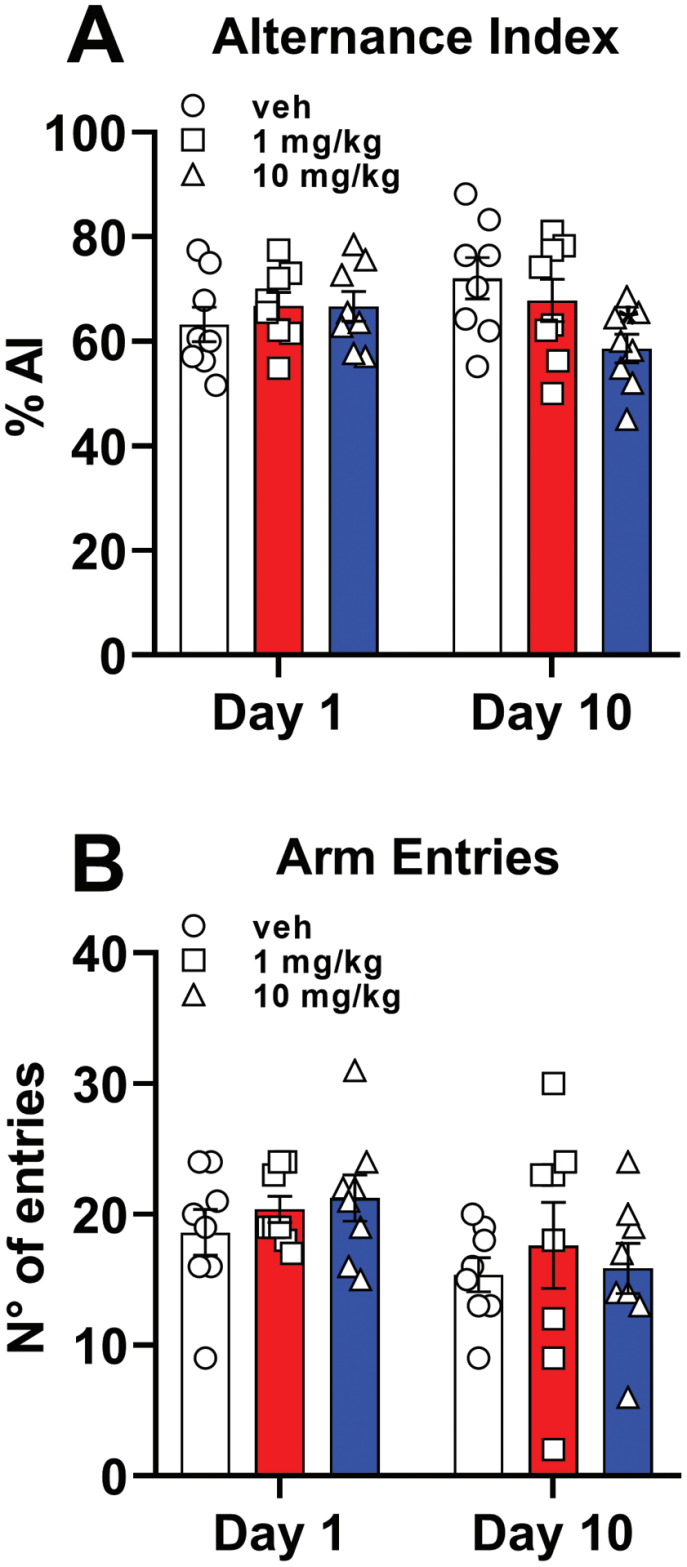
Alternance index (A) and the number of arm entries (B) in the Y maze in rats treated with CE-158 1 mg/kg or 10 mg/kg compared with vehicle (Veh)-treated rats after the first (day 1) and the last (day 10) treatment. Values are expressed as mean ± SEM of 8 rats/group. *P < .05 compared with vehicle-treated rats (RM 2-way ANOVA followed by Bonferroni’s multiple comparison test).

We also tested the impact of protracted treatment with CE-158 on working memory and behavioral flexibility, 2 dopamine-related executive functions that are altered in several psychopathological conditions, from psychosis to drug abuse (Waltz, 2017; Potvin et al., 2018), and whose alteration represents a classical sign of hypofrontality (i.e., a condition characterized by impulsive-compulsive behaviors, impairments in working memory and behavioral flexibility, stereotypies, and perseverations). We took advantage of the Y-maze test, a mPFC-related behavioral procedure. As reported in Figure 6A, CE-158 given to rats at the doses of 1 or 10 mg/kg, either after single or 10 days of repeated administration, did not affect the alternance of arm entries compared with vehicle (2-way ANOVA, treatment F_(2,21)_ = 1.079, *P* = .358). However, post hoc comparisons revealed a significant difference between vehicle- and 10-mg/kg CE-158–treated rats (*P* < .05) in the alternance index after repeated exposure, although with no difference between acute and chronic administration for both treatment groups. Similarly, as reported in Figure 6B, the total number of arm entries did not differ between the 3 treatment groups on either day 1 or after 10 days of treatment (2-way ANOVA, F_(2,21)_ = 0.277, *P* = .761), further confirming that CE-158 does not impair or alter general locomotion/exploration in rats. The results obtained with the Y-maze suggest that a 10-day chronic treatment with CE-158 does not alter or disrupt exploration, flexibility, and/or working memory.

### Grooming

**Figure 7.**
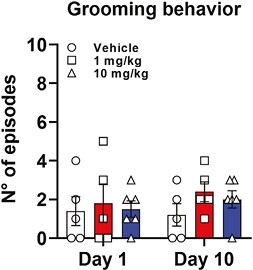
Grooming behavior in rats treated with CE-158 1 mg/kg (n = 5) or 10 mg/kg (n = 6) compared with vehicle-treated rats (n = 5) after the first (day 1) and the last (day 10) treatment. Values are expressed as mean ± SEM (RM 2-way ANOVA: not significant).

Excessive grooming is considered a putative sign of stereotyped and compulsive behavior (Berridge et al., 2005; Feusner et al., 2009) when associated with conditions of chronically elevated dopamine activity (Berridge et al., 2005; Taylor et al., 2010), even related to DAT dysfunctions (Sanna et al., 2020). On the other hand, medium to high doses of psychostimulants such as cocaine or amphetamines totally suppress spontaneous grooming behavior in rats (Antoniou et al., 1998). Hence, we assessed the frequency of grooming behavior after acute and chronic treatment with CE-158 to assess potential effects of our treatment on this behavioral response. As shown in Figure 7, no significant differences were observed in the number of grooming episodes between rats treated with CE-158 1 mg/kg or 10 mg/kg and vehicle-treated rats (2-way ANOVA, F_(2,13)_ = 0.881, *P* = .437) nor after acute or chronic treatment (2-way ANOVA, F_(1,13)_ = 0.312, *P* = .586).

## DISCUSSION

Here, we show for the first time, to our knowledge, that systemic administration of the new modafinil analog CE-158 significantly increases dopamine neurotransmission in the mPFC of male rats. The effect is dose dependent, with a peak at around 30 minutes after treatment (about 30% and 75% above basal values for the doses of 1 and 10 mg/kg, respectively) and lasts for at least 120 minutes. The extent and time course of increased extracellular dopamine levels is similar to that observed with other DAT inhibitors like modafinil (Rowley et al., 2014), CE-123 (Sagheddu et al., 2020), MK-26 (Kouhnavardi et al., 2022), GBR12909 (Weikop et al., 2007), and methylphenidate (Berridge et al., 2006), although with the latter a greater effect was observed at comparable doses (Bymaster et al., 2002; Marsteller et al., 2002; Rowley et al., 2014). These results are also confirmed by electrophysiology experiments, which are consistent with an increased dopamine activity following drug treatment. In particular, the functional sampling showed that the low dose of CE-158 in a 10 day-chronic regimen is sufficient to activate pyramidal neurons in the mPFC. This is in agreement with our previous experiments following acute treatment with the same dose in both young and aged rats (Lubec et al., 2021) and with an acute injection of methylphenidate either i.v. or locally injected in the PFC (Gronier, 2011). On the contrary, another selective dopamine uptake inhibitor, GBR12909, was not found to activate pyramidal neurons in the mPFC when administered i.v. up to 6 mg/kg (Gronier, 2011), possibly due to a different binding mechanism to the transporter (Schmitt and Reith, 2011).

The increased average firing frequency, resulting from the prolonged CE-158 low-dose treatment, suggests an additional long-term influence on the mPFC, which could be related to the behavioral output (see below). In this brain region, dopamine exerts a modulatory action by acting at different temporal and spatial scale through the interaction with D1-like and D2-like receptors, directly modulating glutamatergic synapses at pyramidal neurons and indirectly on the activity of inhibitory interneurons (Lohani et al., 2019). An optimal dopaminergic tone influences processes such as working memory, attention, and behavioral flexibility (Ott and Nieder, 2019). The drug possibly impacts pyramidal cells, being GABA interneurons not biased by different doses. Interneurons are key within local cortical networks and specifically in the mPFC, where they coordinate several cognitive tasks, ranging from learning and memory (Kupferschmidt et al., 2022) to attention guidance (Kim et al., 2016), and related behaviors (Kvitsiani et al., 2013). Because psychostimulants have been associated with mPFC microcircuit maladaptation in several physiopathological conditions (Lapish et al., 2015; Bisagno et al., 2016; Kuiper et al., 2017), the interneuron steadiness despite chronic treatment with CE-158 is relevant concerning the drug security profile.

The i.v. dose-response curve at the end of the 10-day treatment showed an increased neuronal firing frequency in the mPFC of pretreated rats, irrespective of the low or high CE-158 dose for pretretment, as compared with the maximal firing induced in vehicle-pretreated rats. This further indicates the effectiveness of the chronic vs the acute regimen to sustain the activation of the mPFC.

These functional results are also corroborated by the behavioral findings indicating the ability of the compound to elicit exploratory behavior (i.e., rearing) to a similar extent after both acute and chronic treatment without any sign of tolerance to its pro-active effects, as well as its inability to induce any behavioral alterations typical of chronic psychostimulants (i.e., amphetamines and cocaine). Exploratory behavior and rearing, in particular, can be seen as a reliable discriminant for assessing the presence of cognitive enhancement by a given compound (i.e., increase in wakefulness, vigilance, and attention) without signs of excessive dopamine activity (Wood et al., 2014; Minassian et al., 2016). It is well known that sustained dopaminergic stimulation occurring with psychostimulants can lead to a profound imbalance in the functioning of the mesocorticolimbic circuit, in particular, an excessive activity of the mesolimbic pathway together with hypoactivity of the mesocortical one, with a significant decrease of dopamine-related function in the PFC (Jentsch et al., 2000; Kalivas, 2009; Goto et al., 2010; Hui and Beier, 2022). In preclinical models, alterations in mesocorticolimbic circuitry are reflected by behavioral signs such as changes in locomotor activity (i.e., behavioral sensitization), alterations in sensorimotor gating, impairments in exploratory behavior and working memory, and loss of behavioral flexibility, which reflect the presence of cognitive deficits and/or addiction-like states (Wood et al., 2014).

Our results indicate that neither single nor repeated administration of CE-158 at 1 or 10 mg/kg induced significant increase in locomotor activity. More importantly, our chronic treatment of 10 days did not induce behavioral sensitization. Behavioral sensitization is considered a typical sign of the functional alterations induced by chronic psychostimulants at the level of the mesocorticolimbic circuit. It reflects, in particular, a maladaptive imbalance between the activity of pyramidal glutamatergic neurons and other neural populations in the PFC, dopamine projections from the VTA and GABA interneurons, and it is believed to underlie the chronicization of the addictive state (Steketee, 2003).

A similar lack of effects was also observed in the PPI test evaluating sensorimotor gating. Sensorimotor gating provides a precognitive attentional filter that prevents sensory overload to ensure the proper processing of relevant sensory information, allowing correct cognitive functioning and behavioral responses to environmental stimuli (Braff and Geyer, 1990). This function is sensitive to several psychotropic drugs, particularly dopaminomimetics (Cáceda et al., 2012). One of the brain areas thought to play a key role in sensorimotor gating is the PFC (Swerdlow et al., 2001). Accordingly, PFC dysfunctions, such as those observed in schizophrenia or drug addiction, can lead to impairments of the PPI (Hazlett and Buchsbaum, 2001; Day-Wilson et al., 2006). Our results indicate that CE-158 is ineffective in altering or disrupting sensorimotor gating after acute or prolonged administration.

Spontaneous alternance in the Y maze is considered a general index of exploration, behavioral flexibility, and working memory (Kraeuter et al., 2019), and the PFC is involved in all these functions (Lalonde, 2002). Psychostimulants such as amphetamines affect spontaneous behavioral alternance in the Y maze both in mice (Cherng et al., 2007) and rats (Seyedhosseini Tamijani et al., 2018), increasing perseverative and stereotyped behavior and impairing working memory. Similar alterations can also be observed in DAT knockout rats (Leo et al., 2018; Sanna et al., 2020), a transgenic model of chronic hyperdopaminergia. Here, we did not observe any side effect of the acute or chronic treatment with CE-158 on exploration, working memory, or behavioral flexibility as assessed through the Y-maze test. Accordingly, no difference has been detected between vehicle-treated and CE-158-treated rats in the behavioral alternance index nor in the number of arm entries. A slight, though not significant, increase in the number of arm entries was observed following the acute administration of the compound at the high dose. This result parallels the slight but nonsignificant increase in locomotor activity observed after the acute treatment, possibly reflecting a stimulatory effect on exploration, as supported by the data obtained on the rearing behavior. Likewise, these results are in agreement with the findings that CE-158 enhanced behavioral flexibility and recovered rats from scopolamine-induced impairments in behavioral flexibility as assessed by the attentional set shifting task (Lubec et al., 2021).

Finally, as excessive grooming is a reliable sign of dopamine-induced stereotyped/compulsive behavior (Hollingsworth and Mueller, 1988; Ukai et al., 1992; Antoniou et al., 1998; Berridge et al., 2005; Feusner et al., 2009; Taylor et al., 2010), even directly related to DAT dysfunctions (Sanna et al., 2020), the lack of any significant effect in grooming behavior by CE-158 suggests that at the conditions used in this study the drug is devoid of compulsive-like effects and confirms its inability to induce stereotyped behavior.

The present research has some limits. Firstly, functional changes on density and distribution of dopamine and/or glutamic acid receptors in the mPFC, have not been yet investigated, though this information can be useful in shedding light on the molecular effects of acute and chronic CE-158 (Gonzales et al., 2019). Secondly, our study has been conducted only in males, although a broad literature points out the key role of sex/gender differences in chronic use of psychostimulants both at recreational and clinical settings (Dafny and Yang, 2006; Daiwile et al., 2022; Pisanu et al., 2022). Finally, a differential impact of the anesthetics used for the microdialysis and electrophysiology experiments (isoflurane vs chloral hydrate) cannot be completely ruled out. Further studies are needed to examine the translational potential and safety profile of CE-158.

Nonetheless, our data show that CE-158 dose-dependently elicits responsiveness to environmental probing both after acute and repeated administration without inducing behavioral sensitization in consequence of chronic treatment or compulsive-like behaviors. Moreover, at the doses used, we did not detect any behavioral correlates of psychotic-like signs, such as alterations in sensorimotor gating or in behavioral flexibility and working memory, which have been repeatedly attributed to other psychostimulants (Cherland and Fitzpatrick, 1999). Accordingly, the electrophysiological and neurochemical results support the view that this compound potentiates prefrontal activity without any sign that could resemble the modifications induced by chronic psychostimulants (Bisagno et al., 2016). In conclusion, the novel atypical and selective DAT inhibitor CE-158 can be considered as a promising pro-cognitive compound and contributes to assessing its preclinical safety profile also under repeated administration for further translational/clinical testing.

## Supplementary Material

pyad056_suppl_Supplementary_Figures_S1

## Data Availability

The data underlying this article will be shared on reasonable request to the corresponding author.

## References

[CIT0001] Anastasiades PG , Carter AG (2021) Circuit organization of the rodent medial prefrontal cortex. Trends Neurosci 44:550–563. 33972100 10.1016/j.tins.2021.03.006 PMC8222144

[CIT0002] Angioni L , Cocco C, Ferri G-L, Argiolas A, Melis MR, Sanna F (2016) Involvement of nigral oxytocin in locomotor activity: a behavioral, immunohistochemical and lesion study in male rats. Horm Behav 83:23–38. 27189764 10.1016/j.yhbeh.2016.05.012

[CIT0003] Antoniou K , Kafetzopoulos E, Papadopoulou-Daifoti Z, Hyphantis T, Marselos M (1998) D-amphetamine, cocaine and caffeine: a comparative study of acute effects on locomotor activity and behavioural patterns in rats. Neurosci Biobehav Rev 23:189–196. 9884112 10.1016/s0149-7634(98)00020-7

[CIT0004] Arenas MC , Aguilar MA, Montagud-Romero S, Mateos-García A, Navarro-Francés CI, Miñarro J, Rodríguez-Arias M (2016) Influence of the novelty-seeking endophenotype on the rewarding effects of psychostimulant drugs in animal models. Curr Neuropharmacol 14:87–100. 26391743 10.2174/1570159X13666150921112841 PMC4787288

[CIT0005] Au-Young SM , Shen H, Yang CR (1999) Medial prefrontal cortical output neurons to the ventral tegmental area (VTA) and their responses to burst-patterned stimulation of the VTA: neuroanatomical and in vivo electrophysiological analyses. Synapse 34:245–255. 10529719 10.1002/(SICI)1098-2396(19991215)34:4<245::AID-SYN1>3.0.CO;2-D

[CIT0006] Battleday RM , Brem A-K (2015) Modafinil for cognitive neuroenhancement in healthy non-sleep-deprived subjects: a systematic review. Eur Neuropsychopharmacol 25:1865–1881. 26381811 10.1016/j.euroneuro.2015.07.028

[CIT0007] Beaulieu J-M , Gainetdinov RR, Gainetdinov RR (2011) The physiology, signaling, and pharmacology of dopamine receptors. Pharmacol Rev 63:182–217. 21303898 10.1124/pr.110.002642

[CIT0008] Berridge CW , Devilbiss DM, Andrzejewski ME, Arnsten AF, Kelley AE, Schmeichel B, Hamilton C, Spencer RC (2006) Methylphenidate preferentially increases catecholamine neurotransmission within the prefrontal cortex at low doses that enhance cognitive function. Biol Psychiatry 60:1111–1120. 16806100 10.1016/j.biopsych.2006.04.022

[CIT0009] Berridge KC , Aldridge JW, Houchard KR, Zhuang X (2005) Sequential super-stereotypy of an instinctive fixed action pattern in hyper-dopaminergic mutant mice: a model of obsessive compulsive disorder and Tourette’s. BMC Biol 3:4. 15710042 10.1186/1741-7007-3-4 PMC552313

[CIT0010] Bisagno V , González B, Urbano FJ (2016) Cognitive enhancers versus addictive psychostimulants: the good and bad side of dopamine on prefrontal cortical circuits. Pharmacol Res 109:108–118. 26826399 10.1016/j.phrs.2016.01.013

[CIT0011] Braff DL , Geyer MA (1990) Sensorimotor gating and schizophrenia. Human and animal model studies. Arch Gen Psychiatry 47:181–188. 2405807 10.1001/archpsyc.1990.01810140081011

[CIT0012] Brühl AB , d’Angelo C, Sahakian BJ (2019) Neuroethical issues in cognitive enhancement: Modafinil as the example of a workplace drug? Brain Neurosci Adv 3:2398212818816018. 32166175 10.1177/2398212818816018 PMC7058249

[CIT0013] Bymaster FP , Katner JS, Nelson DL, Hemrick-Luecke SK, Threlkeld PG, Heiligenstein JH, Morin SM, Gehlert DR, Perry KW (2002) Atomoxetine increases extracellular levels of norepinephrine and dopamine in prefrontal cortex of rat: a potential mechanism for efficacy in attention deficit/hyperactivity disorder. Neuropsychopharmacology 27:699–711. 12431845 10.1016/S0893-133X(02)00346-9

[CIT0014] Cáceda R , Binder EB, Kinkead B, Nemeroff CB (2012) The role of endogenous neurotensin in psychostimulant-induced disruption of prepulse inhibition and locomotion. Schizophr Res 136:88–95. 22104138 10.1016/j.schres.2011.10.013 PMC3595536

[CIT0015] Carton L , Niot C, Kyheng M, Petrault M, Laloux C, Potey C, Lenski M, Bordet R, Deguil J (2021) Lack of direct involvement of a diazepam long-term treatment in the occurrence of irreversible cognitive impairment: a pre-clinical approach. Transl Psychiatry 11:612. 34857741 10.1038/s41398-021-01718-8 PMC8640018

[CIT0016] Castellanos FX , Tannock R (2002) Neuroscience of attention-deficit/hyperactivity disorder: the search for endophenotypes. Nat Rev Neurosci 3:617–628. 12154363 10.1038/nrn896

[CIT0017] Cherland E , Fitzpatrick R (1999) Psychotic side effects of psychostimulants: a 5-year review. Canadian J Psychiatry 44:811–813. 10.1177/070674379904400810 10566114

[CIT0018] Cherng CG , Tsai C-W, Tsai Y-P, Ho M-C, Kao S-F, Yu L (2007) Methamphetamine-disrupted sensory processing mediates conditioned place preference performance. Behav Brain Res 182:103–108. 17574689 10.1016/j.bbr.2007.05.010

[CIT0019] Connors BW , Gutnick MJ (1990) Intrinsic firing patterns of diverse neocortical neurons. Trends Neurosci 13:99–104. 1691879 10.1016/0166-2236(90)90185-d

[CIT0020] Dafny N , Yang PB (2006) The role of age, genotype, sex, and route of acute and chronic administration of methylphenidate: a review of its locomotor effects. Brain Res Bull 68:393–405. 16459193 10.1016/j.brainresbull.2005.10.005

[CIT0021] Daiwile AP , Jayanthi S, Cadet JL (2022) Sex differences in methamphetamine use disorder perused from pre-clinical and clinical studies: potential therapeutic impacts. Neurosci Biobehav Rev 137:104674. 35452744 10.1016/j.neubiorev.2022.104674 PMC9119944

[CIT0022] Day-Wilson KM , Jones DNC, Southam E, Cilia J, Totterdell S (2006) Medial prefrontal cortex volume loss in rats with isolation rearing-induced deficits in prepulse inhibition of acoustic startle. Neuroscience 141:1113–1121. 16750891 10.1016/j.neuroscience.2006.04.048

[CIT0023] de Natale ER , Niccolini F, Wilson H, Politis M (2018) Molecular imaging of the dopaminergic system in idiopathic Parkinson’s Disease. Int Rev Neurobiol 141:131–172. 30314595 10.1016/bs.irn.2018.08.003

[CIT0024] Ebner K , Sartori SB, Murau R, Kopel F, Kalaba P, Dragačević V, Leban JJ, Singewald N, Engelmann M, Lubec G (2022) The novel analogue of Modafinil CE-158 protects social memory against interference and triggers the release of dopamine in the nucleus accumbens of mice. Biomolecules 12:506. 35454095 10.3390/biom12040506 PMC9033101

[CIT0025] Everitt BJ , Wolf ME (2002) Psychomotor stimulant addiction: a neural systems perspective. J Neurosci 22:3312–3320. 11978805 10.1523/JNEUROSCI.22-09-03312.2002 PMC6758398

[CIT0026] Feusner JD , Hembacher E, Phillips KA (2009) The mouse who couldn’t stop washing: pathologic grooming in animals and humans. CNS Spectr 14:503–513. 19890232 10.1017/s1092852900023567 PMC2853748

[CIT0027] Fiorentini A , Cantù F, Crisanti C, Cereda G, Oldani L, Brambilla P (2021) Substance-induced psychoses: an updated literature review. Front Psychiatry 12:694863. 35002789 10.3389/fpsyt.2021.694863 PMC8732862

[CIT0028] Gainetdinov RR , Caron MG (2003) Monoamine transporters: from genes to behavior. Annu Rev Pharmacol Toxicol 43:261–284. 12359863 10.1146/annurev.pharmtox.43.050802.112309

[CIT0029] German CL , Baladi MG, McFadden LM, Hanson GR, Fleckenstein AE (2015) Regulation of the dopamine and vesicular monoamine transporters: pharmacological targets and implications for disease. Pharmacol Rev 67:1005–1024. 26408528 10.1124/pr.114.010397 PMC4630566

[CIT0030] Geyer MA , Krebs-Thomson K, Braff DL, Swerdlow NR (2001) Pharmacological studies of prepulse inhibition models of sensorimotor gating deficits in schizophrenia: a decade in review. Psychopharmacology (Berl) 156:117–154. 11549216 10.1007/s002130100811

[CIT0031] González B , Torres OV, Jayanthi S, Gomez N, Sosa MH, Bernardi A, Urbano FJ, García-Rill E, Cadet JL, Bisagno V (2019) The effects of single-dose injections of modafinil and methamphetamine on epigenetic and functional markers in the mouse medial prefrontal cortex: potential role of dopamine receptors. Prog Neuropsychopharmacol Biol Psychiatry 88:222–234. 30056065 10.1016/j.pnpbp.2018.07.019 PMC8424782

[CIT0032] Goto Y , Yang CR, Otani S (2010) Functional and dysfunctional synaptic plasticity in prefrontal cortex: roles in psychiatric disorders. Biol Psychiatry 67:199–207. 19833323 10.1016/j.biopsych.2009.08.026

[CIT0033] Gronier B (2011) In vivo electrophysiological effects of methylphenidate in the prefrontal cortex: involvement of dopamine D1 and alpha 2 adrenergic receptors. Eur Neuropsychopharmacol 21:192–204. 21146374 10.1016/j.euroneuro.2010.11.002

[CIT0034] Hazlett EA , Buchsbaum MS (2001) Sensorimotor gating deficits and hypofrontality in schizophrenia. Front Biosci 6:D1069–D1072. 11532605 10.2741/hazlett

[CIT0035] Hollingsworth EM , Mueller K (1988) Patterns of locomotor and stereotypic behavior during continuous amphetamine administration in rats. Pharmacol Biochem Behav 30:535–537. 3174785 10.1016/0091-3057(88)90493-5

[CIT0036] Howland JG , Ito R, Lapish CC, Villaruel FR (2022) The rodent medial prefrontal cortex and associated circuits in orchestrating adaptive behavior under variable demands. Neurosci Biobehav Rev 135:104569. 35131398 10.1016/j.neubiorev.2022.104569 PMC9248379

[CIT0037] Hui M , Beier KT (2022) Defining the interconnectivity of the medial prefrontal cortex and ventral midbrain. Front Mol Neurosci 15:971349. 35935333 10.3389/fnmol.2022.971349 PMC9354837

[CIT0038] Jentsch JD , Roth RH, Taylor JR (2000) Role for dopamine in the behavioral functions of the prefrontal corticostriatal system: implications for mental disorders and psychotropic drug action. Prog Brain Res 126:433–453. 11105661 10.1016/S0079-6123(00)26028-7

[CIT0039] Kalaba P , et al. (2020) Structure-activity relationships of novel thiazole-based modafinil analogues acting at monoamine transporters. J Med Chem 63:391–417. 31841637 10.1021/acs.jmedchem.9b01938

[CIT0040] Kalivas PW (2009) Perspective: the manifest destiny of cocaine research. Neuropsychopharmacology 34:1089–1090. 19194378 10.1038/npp.2009.9

[CIT0041] Kim H , Ährlund-Richter S, Wang X, Deisseroth K, Carlén M (2016) Prefrontal parvalbumin neurons in control of attention. Cell 164:208–218. 26771492 10.1016/j.cell.2015.11.038 PMC4715187

[CIT0042] Klein MO , Battagello DS, Cardoso AR, Hauser DN, Bittencourt JC, Correa RG (2019) Dopamine: functions, signaling, and association with neurological diseases. Cell Mol Neurobiol 39:31–59. 30446950 10.1007/s10571-018-0632-3 PMC11469830

[CIT0043] Kouhnavardi S , et al (2022) A novel and selective dopamine transporter inhibitor, (S)-MK-26, promotes hippocampal synaptic plasticity and restores effort-related motivational dysfunctions. Biomolecules 12:881. 35883437 10.3390/biom12070881 PMC9312958

[CIT0044] Kraeuter A-K , Guest PC, Sarnyai Z (2019) The Y-maze for assessment of spatial working and reference memory in mice. Methods Mol Biol (Clifton, N.J.) 1916:105–111. 10.1007/978-1-4939-8994-2_10 30535688

[CIT0045] Kristofova M , Aher YD, Ilic M, Radoman B, Kalaba P, Dragacevic V, Aher NY, Leban J, Korz V, Zanon L, Neuhaus W, Wieder M, Langer T, Urban E, Sitte HH, Hoeger H, Lubec G, Aradska J (2018) A daily single dose of a novel modafinil analogue CE-123 improves memory acquisition and memory retrieval. Behav Brain Res 343:83–94. 29410048 10.1016/j.bbr.2018.01.032

[CIT0046] Kuhar MJ , Sanchez-Roa PM, Wong DF, Dannals RF, Grigoriadis DE, Lew R, Milberger M (1990) Dopamine transporter: biochemistry, pharmacology and imaging. Eur Neurol 30:15–20. 2178937 10.1159/000117169

[CIT0047] Kuiper LB , Frohmader KS, Coolen LM (2017) Maladaptive sexual behavior following concurrent methamphetamine and sexual experience in male rats is associated with altered neural activity in frontal cortex. Neuropsychopharmacology 42:2011–2020. 28051103 10.1038/npp.2017.1 PMC5561340

[CIT0048] Kupferschmidt DA , Cummings KA, Joffe ME, MacAskill A, Malik R, Sánchez-Bellot C, Tejeda HA, Yarur Castillo H (2022) Prefrontal interneurons: populations, pathways, and plasticity supporting typical and disordered cognition in rodent models. J Neurosci 42:8468–8476. 36351822 10.1523/JNEUROSCI.1136-22.2022 PMC9665918

[CIT0049] Kvitsiani D , Ranade S, Hangya B, Taniguchi H, Huang JZ, Kepecs A (2013) Distinct behavioural and network correlates of two interneuron types in prefrontal cortex. Nature 498:363–366. 23708967 10.1038/nature12176 PMC4349584

[CIT0050] Lalonde R (2002) The neurobiological basis of spontaneous alternation. Neurosci Biobehav Rev 26:91–104. 11835987 10.1016/s0149-7634(01)00041-0

[CIT0051] Lapish CC , Balaguer-Ballester E, Seamans JK, Phillips AG, Durstewitz D (2015) Amphetamine exerts dose-dependent changes in prefrontal cortex attractor dynamics during working memory. J Neurosci 35:10172–10187. 26180194 10.1523/JNEUROSCI.2421-14.2015 PMC4502258

[CIT0052] Lapworth K , Dawe S, Davis P, Kavanagh D, Young R, Saunders J (2009) Impulsivity and positive psychotic symptoms influence hostility in methamphetamine users. Addict Behav 34:380–385. 19097704 10.1016/j.addbeh.2008.11.014

[CIT0053] Leo D , et al. (2018) Pronounced hyperactivity, cognitive dysfunctions, and BDNF dysregulation in dopamine transporter knock-out rats. J Neurosci 38:1959–1972. 29348190 10.1523/JNEUROSCI.1931-17.2018 PMC5824739

[CIT0054] Lohani S , Martig AK, Deisseroth K, Witten IB, Moghaddam B (2019) Dopamine modulation of prefrontal cortex activity is manifold and operates at multiple temporal and spatial scales. Cell Rep 27:99–114.e6. 30943418 10.1016/j.celrep.2019.03.012 PMC11884507

[CIT0055] Lubec J , et al (2021) Reinstatement of synaptic plasticity in the aging brain through specific dopamine transporter inhibition. Mol Psychiatry 26:7076–7090. 34244620 10.1038/s41380-021-01214-x

[CIT0056] Lubec J , et al (2023) Low-affinity/high-selectivity dopamine transport inhibition sufficient to rescue cognitive functions in the aging rat. Biomolecules 13:467. 36979402 10.3390/biom13030467 PMC10046369

[CIT0057] Maia TV , Frank MJ (2017) An integrative perspective on the role of dopamine in schizophrenia. Biol Psychiatry 81:52–66. 27452791 10.1016/j.biopsych.2016.05.021 PMC5486232

[CIT0058] Marsteller DA , Gerasimov MR, Schiffer WK, Geiger JM, Barnett CR, Schaich Borg J, Scott S, Ceccarelli J, Volkow ND, Molina PE, Alexoff DL, Dewey SL (2002) Acute handling stress modulates methylphenidate-induced catecholamine overflow in the medial prefrontal cortex. Neuropsychopharmacology 27:163–170. 12093590 10.1016/S0893-133X(02)00288-9

[CIT0059] Melis MR , Sanna F, Argiolas A (2022) Dopamine, erectile function and male sexual behavior from the past to the present: a review. Brain Sci 12:826. 35884633 10.3390/brainsci12070826 PMC9312911

[CIT0060] Mena A , Ruiz-Salas JC, Puentes A, Dorado I, Ruiz-Veguilla M, De la Casa LG (2016) Reduced prepulse inhibition as a biomarker of schizophrenia. Front Behav Neurosci 10:202. 27803654 10.3389/fnbeh.2016.00202 PMC5067522

[CIT0061] Mena A , López S, Ruiz-Salas JC, Fernández A, Pérez-Díaz FJ, López JC (2021) Sensitivity to amphetamine in prepulse inhibition response requires a mature medial prefrontal cortex. Behav Neurosci 135:32–38. 33734732 10.1037/bne0000458

[CIT0062] Mereu M , Bonci A, Newman AH, Tanda G (2013) The neurobiology of modafinil as an enhancer of cognitive performance and a potential treatment for substance use disorders. Psychopharmacology (Berl) 229:415–434. 23934211 10.1007/s00213-013-3232-4 PMC3800148

[CIT0063] Mereu M , Chun LE, Prisinzano TE, Newman AH, Katz JL, Tanda G (2017) The unique psychostimulant profile of (±)-modafinil: investigation of behavioral and neurochemical effects in mice. Eur J Neurosci 45:167–174. 27545285 10.1111/ejn.13376 PMC5604337

[CIT0064] Minassian A , Young JW, Cope ZA, Henry BL, Geyer MA, Perry W (2016) Amphetamine increases activity but not exploration in humans and mice. Psychopharmacology (Berl) 233:225–233. 26449721 10.1007/s00213-015-4098-4 PMC4703551

[CIT0065] Nestler EJ , Barrot M, DiLeone RJ, Eisch AJ, Gold SJ, Monteggia LM (2002) Neurobiology of depression. Neuron 34:13–25. 11931738 10.1016/s0896-6273(02)00653-0

[CIT0066] Nikiforuk A , Kalaba P, Ilic M, Korz V, Dragačević V, Wackerlig J, Langer T, Höger H, Golebiowska J, Popik P, Lubec G (2017) A novel dopamine transporter inhibitor CE-123 improves cognitive flexibility and maintains impulsivity in healthy male rats. Front Behav Neurosci 11:222. 29230168 10.3389/fnbeh.2017.00222 PMC5711856

[CIT0067] Noli B , Sanna F, Brancia C, D’Amato F, Manconi B, Vincenzoni F, Messana I, Melis MR, Argiolas A, Ferri G-L, Cocco C (2017) Profiles of VGF peptides in the rat brain and their modulations after phencyclidine treatment. Front Cell Neurosci 11:158. 28626390 10.3389/fncel.2017.00158 PMC5454051

[CIT0068] Ott T , Nieder A (2019) Dopamine and cognitive control in prefrontal cortex. Trends Cogn Sci 23:213–234. 30711326 10.1016/j.tics.2018.12.006

[CIT0069] Paxinos G , Watson C (2004) The rat brain in stereotaxic coordinates. 6th ed. Elsevier Academic Press.

[CIT0070] Pisanu A , Lo Russo G, Talani G, Bratzu J, Siddi C, Sanna F, Diana M, Porcu P, De Luca MA, Fattore L (2022) Effects of the phenethylamine 2-Cl-4,5-MDMA and the synthetic cathinone 3,4-MDPHP in adolescent rats: focus on sex differences. Biomedicines 10:2336. 36289598 10.3390/biomedicines10102336 PMC9598216

[CIT0071] Pisula W , Siegel J (2005) Exploratory behavior as a function of environmental novelty and complexity in male and female rats. Psychol Rep 97:631–638. 16342593 10.2466/pr0.97.2.631-638

[CIT0072] Potvin S , Pelletier J, Grot S, Hébert C, Barr AM, Lecomte T (2018) Cognitive deficits in individuals with methamphetamine use disorder: a meta-analysis. Addict Behav 80:154–160. 29407687 10.1016/j.addbeh.2018.01.021

[CIT0073] Repantis D , Schlattmann P, Laisney O, Heuser I (2010) Modafinil and methylphenidate for neuroenhancement in healthy individuals: a systematic review. Pharmacol Res 62:187–206. 20416377 10.1016/j.phrs.2010.04.002

[CIT0074] Robbins TW , Arnsten AFT (2009) The neuropsychopharmacology of fronto-executive function: monoaminergic modulation. Annu Rev Neurosci 32:267–287. 19555290 10.1146/annurev.neuro.051508.135535 PMC2863127

[CIT0075] Rotolo RA , Dragacevic V, Kalaba P, Urban E, Zehl M, Roller A, Wackerlig J, Langer T, Pistis M, De Luca MA, Caria F, Schwartz R, Presby RE, Yang J-H, Samels S, Correa M, Lubec G, Salamone JD (2019) The novel atypical dopamine uptake inhibitor (S)-CE-123 partially reverses the effort-related effects of the dopamine depleting agent tetrabenazine and increases progressive ratio responding. Front Pharmacol 10:682. 31316379 10.3389/fphar.2019.00682 PMC6611521

[CIT0076] Rotolo RA , Kalaba P, Dragacevic V, Presby RE, Neri J, Robertson E, Yang J-H, Correa M, Bakulev V, Volkova NN, Pifl C, Lubec G, Salamone JD (2020) Behavioral and dopamine transporter binding properties of the modafinil analog (S, S)-CE-158: reversal of the motivational effects of tetrabenazine and enhancement of progressive ratio responding. Psychopharmacology (Berl) 237:3459–3470. 32770257 10.1007/s00213-020-05625-6 PMC7572767

[CIT0077] Rowley HL , Kulkarni RS, Gosden J, Brammer RJ, Hackett D, Heal DJ (2014) Differences in the neurochemical and behavioural profiles of lisdexamfetamine methylphenidate and modafinil revealed by simultaneous dual-probe microdialysis and locomotor activity measurements in freely-moving rats. J Psychopharmacol 28:254–269. 24327450 10.1177/0269881113513850

[CIT0078] Sagheddu C , Pintori N, Kalaba P, Dragačević V, Piras G, Lubec J, Simola N, De Luca MA, Lubec G, Pistis M (2020) Neurophysiological and neurochemical effects of the putative cognitive enhancer (S)-CE-123 on mesocorticolimbic dopamine system. Biomolecules 10:779. 32443397 10.3390/biom10050779 PMC7277835

[CIT0079] Sagheddu C , Traccis F, Serra V, Congiu M, Frau R, Cheer JF, Melis M (2021) Mesolimbic dopamine dysregulation as a signature of information processing deficits imposed by prenatal THC exposure. Prog Neuropsychopharmacol Biol Psychiatry 105:110128. 33031862 10.1016/j.pnpbp.2020.110128

[CIT0080] Sanna F , Piludu MA, Corda MG, Melis MR, Giorgi O, Argiolas A (2015) Involvement of dopamine in the differences in sexual behaviour between Roman high and low avoidance rats: an intracerebral microdialysis study. Behav Brain Res 281:177–186. 25497705 10.1016/j.bbr.2014.12.009

[CIT0081] Sanna F , Bratzu J, Piludu MA, Corda MG, Melis MR, Giorgi O, Argiolas A (2017) Dopamine, noradrenaline and differences in sexual behavior between roman high and low avoidance male rats: a microdialysis study in the medial prefrontal cortex. Front Behav Neurosci 11:108. 28638325 10.3389/fnbeh.2017.00108 PMC5461293

[CIT0082] Sanna F , Bratzu J, Serra MP, Leo D, Quartu M, Boi M, Espinoza S, Gainetdinov RR, Melis MR, Argiolas A (2020) Altered sexual behavior in dopamine transporter (DAT) knockout male rats: a behavioral, neurochemical and intracerebral microdialysis study. Front Behav Neurosci 14:58. 32372926 10.3389/fnbeh.2020.00058 PMC7185326

[CIT0083] Schmitt KC , Reith ME (2011) The atypical stimulant and nootropic modafinil interacts with the dopamine transporter in a different manner than classical cocaine-like inhibitors. PLoS One 6:e25790. 22043293 10.1371/journal.pone.0025790 PMC3197159

[CIT0084] Schwaller B , Tetko IV, Tandon P, Silveira DC, Vreugdenhil M, Henzi T, Potier MC, Celio MR, Villa AEP (2004) Parvalbumin deficiency affects network properties resulting in increased susceptibility to epileptic seizures. Mol Cell Neurosci 25:650–663. 15080894 10.1016/j.mcn.2003.12.006

[CIT0085] Secci ME , Mascia P, Sagheddu C, Beggiato S, Melis M, Borelli AC, Tomasini MC, Panlilio LV, Schindler CW, Tanda G, Ferré S, Bradberry CW, Ferraro L, Pistis M, Goldberg SR, Schwarcz R, Justinova Z (2019) Astrocytic mechanisms involving kynurenic acid control Δ9-tetrahydrocannabinol-induced increases in glutamate release in brain reward-processing areas. Mol Neurobiol 56:3563–3575. 30151725 10.1007/s12035-018-1319-y PMC6393222

[CIT0086] Seyedhosseini Tamijani SM , Beirami E, Ahmadiani A, Dargahi L (2018) Effect of three different regimens of repeated methamphetamine on rats’ cognitive performance. Cogn Process 19:107–115. 28948389 10.1007/s10339-017-0839-0

[CIT0087] Shoemaker JM , Saint Marie RL, Bongiovanni MJ, Neary AC, Tochen LS, Swerdlow NR (2005) Prefrontal D1 and ventral hippocampal N-methyl-D-aspartate regulation of startle gating in rats. Neuroscience 135:385–394. 16125865 10.1016/j.neuroscience.2005.06.054 PMC1364454

[CIT0088] Spencer RC , Devilbiss DM, Berridge CW (2015) The cognition-enhancing effects of psychostimulants involve direct action in the prefrontal cortex. Biol Psychiatry 77:940–950. doi:10.1016/j.biopsych.2014.09.013. 25499957 PMC4377121

[CIT0089] Steffensen SC , Svingos AL, Pickel VM, Henriksen SJ (1998) Electrophysiological characterization of GABAergic neurons in the ventral tegmental area. J Neurosci 18:8003–8015. 9742167 10.1523/JNEUROSCI.18-19-08003.1998 PMC6793009

[CIT0090] Steketee JD (2003) Neurotransmitter systems of the medial prefrontal cortex: potential role in sensitization to psychostimulants. Brain Res Brain Res Rev 41:203–228. 12663081 10.1016/s0165-0173(02)00233-3

[CIT0091] Sturman O , Germain P-L, Bohacek J (2018) Exploratory rearing: a context- and stress-sensitive behavior recorded in the open-field test. Stress 21:443–452. 29451062 10.1080/10253890.2018.1438405

[CIT0092] Swerdlow NR , Geyer MA, Braff DL (2001) Neural circuit regulation of prepulse inhibition of startle in the rat: current knowledge and future challenges. Psychopharmacology (Berl) 156:194–215. 11549223 10.1007/s002130100799

[CIT0093] Taylor JL , Rajbhandari AK, Berridge KC, Aldridge JW (2010) Dopamine receptor modulation of repetitive grooming actions in the rat: potential relevance for Tourette syndrome. Brain Res 1322:92–101. 20114036 10.1016/j.brainres.2010.01.052 PMC2858339

[CIT0094] Ukai M , Toyoshi T, Kameyama T (1992) Multidimensional behavioral analyses show dynorphin A-(1-13) modulation of methamphetamine-induced behaviors in mice. Eur J Pharmacol 222:7–12. 1361442 10.1016/0014-2999(92)90455-d

[CIT0095] Volkow ND , Wise RA, Baler R (2017) The dopamine motive system: implications for drug and food addiction. Nat Rev Neurosci 18:741–752. 29142296 10.1038/nrn.2017.130

[CIT0096] Waltz JA (2017) The neural underpinnings of cognitive flexibility and their disruption in psychotic illness. Neuroscience 345:203–217. 27282085 10.1016/j.neuroscience.2016.06.005 PMC5143214

[CIT0097] Weikop P , Kehr J, Scheel-Krüger J (2007) Reciprocal effects of combined administration of serotonin, noradrenaline and dopamine reuptake inhibitors on serotonin and dopamine levels in the rat prefrontal cortex: the role of 5-HT1A receptors. J Psychopharmacol 21:795–804. 17984160 10.1177/0269881107077347

[CIT0098] Wood S , Sage JR, Shuman T, Anagnostaras SG (2014) Psychostimulants and cognition: a continuum of behavioral and cognitive activation. Pharmacol Rev 66:193–221. 24344115 10.1124/pr.112.007054 PMC3880463

