# Supplementary material for: The Atypical Dopamine Transporter Inhibitor CE-158 Enhances Dopamine Neurotransmission in the Prefrontal Cortex of Male Rats: A Behavioral, Electrophysiological, and Microdialysis Study
Source: Int J Neuropsychopharmacol. 2023 Sep 19;26(11):784–95. doi: 10.1093/ijnp/pyad056 (PMC10674083; doi:10.1093/ijnp/pyad056)
Supplement: pyad056_suppl_Supplementary_Figures_S1 [file pyad056_suppl_supplementary_figures_s1.docx]

*Histological verification of microdialysis probe placement in the mPFC*

At the end of the microdialysis experiments the rats were deeply anesthetized with isoflurane and sacrificed by decapitation, the brains were immediately removed from the skull and stored in 4% aqueous formaldehyde for 12-15 days. After this period, 40 µm coronal brain sections were prepared with a freezing microtome, stained with Neutral Red and inspected on a phase contrast microscope. Only rats with the active part of the dialyzing membrane positioned correctly into the mPFC were considered for the statistical evaluation of the results.

| 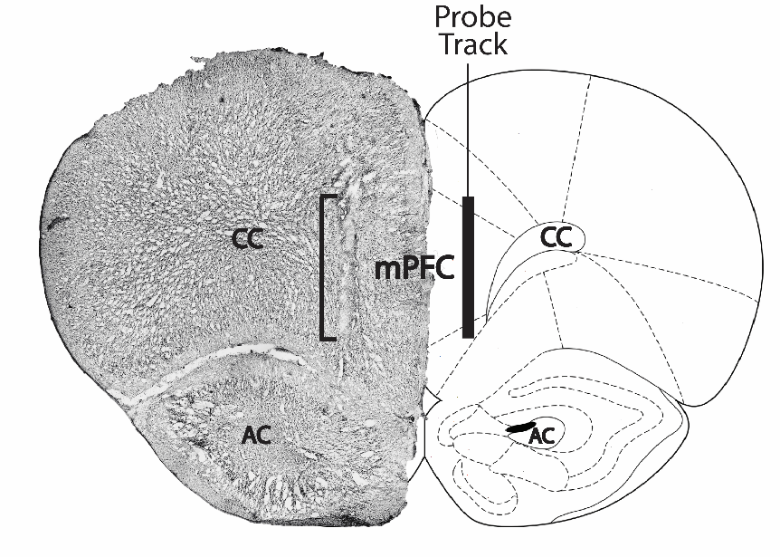 |
| --- |
| *Schematic representation of a coronal section of the rat brain (adapted from Paxinos and Watson, 2004) showing the track of the microdialysis probe in the mPFC of a rat treated with CE-158. The square bracket in the micro-photograph indicates the portion of the histological section showing the active part of the dialyzing membrane of the microdialysis probe. AC = anterior commissura; CC = corpus callosum* |
